# A six-gene-based signature for breast cancer radiotherapy sensitivity estimation

**DOI:** 10.1042/BSR20202376

**Published:** 2020-12-02

**Authors:** Xing Chen, Junjie Zheng, Min ling Zhuo, Ailong Zhang, Zhenhui You

**Affiliations:** 1 Department of General Surgery, Fujian Medical University Provincial Clinical Medical College, Fujian Provincial Hospital, Fujian, 350000, China; 2 Fujian Medical University, Fuzhou, Fujian, 350000, China

**Keywords:** autoencoder, Breast cancer, LASSO Cox regression, radiotherapy sensitivity

## Abstract

Breast cancer (BRCA) represents the most common malignancy among women worldwide with high mortality. Radiotherapy is a prevalent therapeutic for BRCA that with heterogeneous effectiveness among patients. Here, we proposed to develop a gene expression-based signature for BRCA radiotherapy sensitivity estimation. Gene expression profiles of BRCA samples from the Cancer Genome Atlas (TCGA) and International Cancer Genome Consortium (ICGC) were obtained and used as training and independent testing dataset, respectively. Differential expression genes (DEGs) in BRCA samples compared with their paracancerous samples in the training set were identified by using the edgeR Bioconductor package. Univariate Cox regression analysis and LASSO Cox regression method were applied to screen optimal genes for constructing a radiotherapy sensitivity estimation signature. Nomogram combining independent prognostic factors was used to predict 1-, 3-, and 5-year OS of radiation-treated BRCA patients. Relative proportions of tumor infiltrating immune cells (TIICs) calculated by CIBERSORT and mRNA levels of key immune checkpoint receptors was adopted to explore the relation between the signature and tumor immune response. As a result, 603 DEGs were obtained in BRCA tumor samples, six of which were retained and used to construct the radiotherapy sensitivity prediction model. The signature was proved to be robust in both training and testing sets. In addition, the signature was closely related to the immune microenvironment of BRCA in the context of TIICs and immune checkpoint receptors’ mRNA levels. In conclusion, the present study obtained a radiotherapy sensitivity estimation signature for BRCA, which should shed new light in clinical and experimental research.

## Introduction

In terms of incidence, breast cancer (BRCA) is main cancer affecting women [1]. Over 2012–2016, the incidence rate of BRCA increased slightly by 0.3% per year [2]. Mastectomy and breast-sparing surgery for radiotherapy are the most common treatment options [3]. For the patients with early-stage BRCA, accelerated partial breast irradiation (APBI) and intensity-modulated radiotherapy (IMRT) have been introduced as an alternative treatment method of mastectomy [4]. A population-based study showed that breast-conserving surgery plus radiotherapy is at least equivalent to mastectomy with respect to 10-year overall survival [5]. However, radiotherapy may bring side effects to patients at the same time. The estimated absolute risks from modern radiotherapy for BRCA include lung cancer and cardiac mortality, and for long-term smokers, the absolute risks of modern radiotherapy may outweigh the benefits [6]. These results would influence treatment decision making for patients with BRCA.

The decisions for radiotherapy should be tailored on the basis of patient factors, tumor biology, and the prognostic score [7]. Given the predictive markers identify populations of patients who will receive substantial benefit from a specific therapy, genomic assays measuring the expression of multiple genes have been developed to predict response to the treatments in recent years [8]. DeLorenzi et al. reported a 70-gene signature for the treatment decisions in early-stage BRCA and indicated that approximately 46% of women with BRCA with high clinical risk might not require chemotherapy [9]. Although the genotypes of 90 confirmed breast cancer risk variants were not associated with the risk of radiotherapy toxicity up to 5 years following radiotherapy, the individual variants may increase risk [10]. Moreover, microRNA-related DNA repair/cell-cycle genes were also reported to be independently associated with relapse after radiotherapy for early BRCA [11]. Currently, progress in the development of molecular markers to predict the radiation response and necessity of women with BRCA radiation remains slow [12].

We here propose to investigate the potential underlying association between gene expression and BRCA patients’ radiotherapy sensitivity, and ultimately prioritize a radiotherapy sensitivity estimation signature. Estimation roles of the gene expression-based signature were finally demonstrated in both radiotherapy and immune response.

## Materials and methods

### Study population

All the patient information was obtained from public resource. A total of 1217 breast cancer samples were obtained from TCGA (http://tcgaportal.org/), which included 99 paired tumor and paracancerous tissue samples and 1019 unpaired tumor samples. Besides, 1019 breast cancer samples with their complete survival information were also obtained from ICGC (https://icgc.org/). There were 652 and 65 patients from TCGA and ICGC dataset were previously treated with radiation and used as training and testing set, respectively. The clinicopathological characteristics of patients from the training and testing sets were provided in Table 1.

**Table 1:** Clinicopathological characteristics of BRCA radiotherapy samples from TCGA and ICGC database

Parameters	Radiotherapy patients		χ^2^	*P*-value
	TCGA cohort (*N*=652)	ICGC cohort (*N*=65)		
**Age (mean ± SD)**	57.60 ± 9.22	56.97 ± 10.94	/	0.9317
**Pathologic stage**				
**I**	99(15.18%)	26(40%)	6.9987	0.0652
**II**	310(47.55%)	30(46.15%)		
**III**	174(26.69%)	9(13.85%)		
**IV**	9(1.38%)	0(0%)		
**Unknown**	60(9.20%)	0(0%)		
**OS status**				
**Dead**	53(8.13%)	9(13.85%)	0.69046	0.406
**Alive**	539(82.67%)	56(86.15%)		
**Unknown**	60(9.20%)	0(0%)		

### Differential expression analysis

Differential expression analysis between the 99 paired samples was performed by using the edgeR package [13] in R. The gene that with FDR ≤ 0.05 and absolute log2 fold change (FC) > 1 was defined as the differentially expressed gene (DEG).

### Dimensionality reduction through autoencoder neural network algorithm

Autoencoder is an unsupervised learning technique which takes raw data without a label as input and tries to reconstruct it by using a fewer number of bits from the bottleneck layer. In the present study, we proposed to reduce candidate genes for the radiotherapy prediction model by using the autoencoder neural network algorithm through which dimensionality reduction could be carried out. H2O R package (https://cran.r-project.org/web/packages/h2o/) was adapted to perform autoencoder analysis with the gene number in the bottleneck layer specified as 50.

### Construction of the prognostic model

Univariate Cox-regression analysis was performed to screen for genes that were significantly associated with breast cancer’s overall survival (OS). LASSO Cox-regression analysis was further used to construct the prognostic model by glmnet [14] function package in R and calculated the risk score. The risk score was calculated on the basis of the following formula:

$$Risk\;score=\sum_{i=1}^{n}Coef_{i}\times x_{i}$$

*Coef_i_* was the risk coefficient of each factor calculated by the LASSO-Cox model, and x_*i*_ was the expression value of each factor, which referred to the gene expression level in the present study.

### Survival analysis

The survival probability of BRCA patients was assessed by the Kaplan–Meier survival curve using the survival package (https://cran.r-project.org/web/packages/survival/) in R language. A log-rank test was used to assess the difference of OS between different groups with a significant threshold of *P*<0.05.

### Nomogram analysis

Multivariate Cox-regression analysis was applied to determine the independence of associations of several factors, including age, TNM stage, and risk score, and BRCA patients’ OS probability treated with radiotherapy. Nomogram used to estimate BRCA patients’ 1-, 3-, and 5-year OS probability was constructed by incorporating those independent prognostic factors through rms R package (https://cran.r-project.org/web/packages/rms/rms). The calibration curve was plotted to evaluate the deviation between the estimated and actual OS probability.

### Tumor infiltrating immune cell analysis

Tumor infiltrating immune cells (TIICs) is an intrinsic property of all tumors which have been extensively studied and proved to be closely associated with cancers’ clinical performance. In the present study, we used CIBERSORT (https://cibersort.stanford.edu/), a gene expression-based method, to estimate relative proportions of 22 TIICs across those radiation-treated BRCA tumor samples.

### Statistical analysis

Chi-square test was used to compare distributions of samples across different clinicopathological groups except for age between training and testing set. Wilcoxon's method was used as the comparison method for age in the training cohort and testing cohort. The receiver operating characteristic (ROC) analysis [15] was used for evaluating the performance of models. Univariate Cox-regression analysis was adapted to screen OS-related genes. Statistical analysis was applied using R version 3.5.2. The above threshold was *P*<0.05 unless otherwise specified.

## Results

### Differential expression genes in BRCA tumor samples

**Figure 1:**
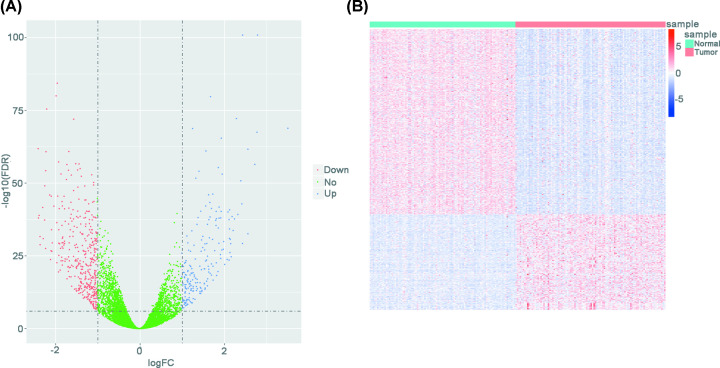
Differential gene expression analysis between paired BRCA tumor and paracancerous tissues from TCGA (**A**) Volcano plot displaying gene differential expression pattern in BRCA tumor tissues compared with paracancerous tissues. Red and blue dot represents down- and up-regulated genes, respectively. Green dots are nondifferential expression genes. (**B**) Heatmap showing differential expression genes’ (DEGs’) mRNA level after *Z*-score normalization in paired BRCA tumor and paracancerous tissues. Horizontal and vertical axis represents genes and samples, respectively.

A total of 603 DEGs including 205 up- and 398 down-regulated ones were identified in BRCA tumor samples compared with their paracancerous samples. Figure 1A illustrated the differential expression pattern of all genes, and Figure 1B showed the *Z*-score normalized mRNA expressions of the 603 DEGs in paired BRCA tumor and paracancerous tissues.

### Autoencoder screened genes

For the apparent correlation among the DEGs, i.e. they were significantly up- or down-regulated in BRCA tumor samples, we applied an autoencoder algorithm, which is a neural network-based learning technique for representation learning, for screening the most representative genes for the following analysis. The input features were expression profiles of the 603 genes in paired BRCA tumor and paracancerous tissues, and the number of layers and gene numbers contained in the bottleneck layer were specified as 5 and 50, respectively. Table 2 provided the finally retained 50 genes along with their relative importance.

**Table 2:** The 50 genes remained through autoencoder algorithm

Gene	Score	Gene	Score
HOXB13	1	FCRL4	0.796
CXADRP3	0.973	LINC00898	0.795
GNGT1	0.941	LOC101928932	0.793
NKX2_2	0.918	TLX1	0.790
POTEC	0.909	MS4A15	0.790
PYDC1	0.897	GOLGA8T	0.787
DSCAM.AS1	0.886	LOC284930	0.781
MAGEA6	0.867	MIR8071_1	0.778
LINC01644	0.866	UBE2E2.AS1	0.775
ABCC13	0.860	EIF4E1B	0.775
VSTM2A_OT1	0.856	ASCL1	0.771
CLPS	0.843	WT1_AS	0.765
C5orf66_AS1	0.842	LINC00052	0.762
EPHA8	0.841	GOLGA6L3	0.762
C8orf34_AS1	0.839	LINC00628	0.761
KISS1R	0.837	OPRPN	0.760
ADAMTS20	0.828	GAL3ST2	0.760
LINC01844	0.827	LINC00466	0.759
DLX2_DT	0.820	ACTL8	0.757
FOXD3_AS1	0.816	LINC01344	0.755
LOC339685	0.814	GPR139	0.750
WT1	0.809	RTBDN	0.750
MAGEA3	0.807	TMEM270	0.748
TACR3	0.803	LOC101928978	0.747
LHFPL5	0.803	PRAC2	0.744

### Radiotherapy sensitivity prediction model

**Figure 2:**
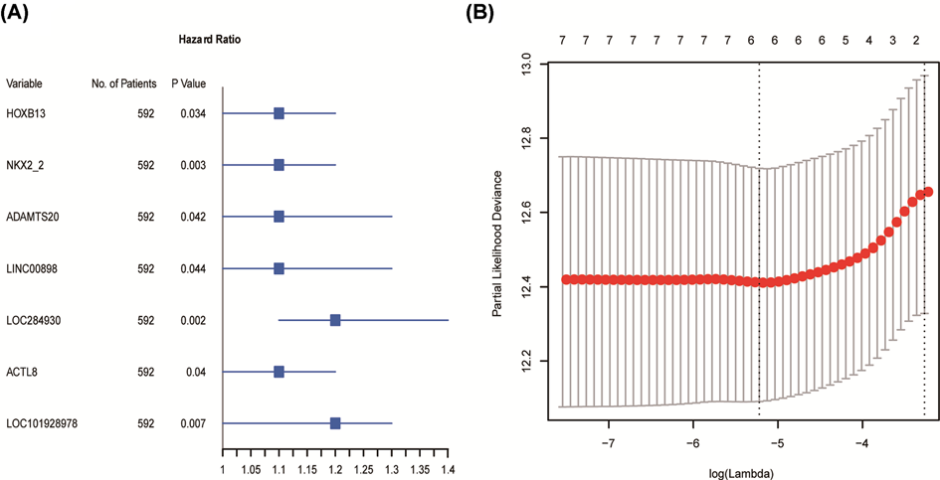
Construction of BRCA radiotherapy sensitivity prediction model (**A**) Seven genes that significantly associated with overall survival (OS) of BRCA patients in radiotherapy group. Square data indicate the hazard ratios (HRs) with error bars are 95% confidence intervals (CI). (**B**) Selection of optimal variables, i.e. genes here, for constructing prediction model through LASSO Cox-regression method. The optimal variable number was determined by the vertical dashed line at which the Partial Likelihood Deviance (PLD) was lowest.

Univariate Cox-regression analysis identified seven out of the 50 autoencoder remained genes including *HOXB13, NKX2-2, ADAMTS20, LINC00898, LOC284930, ACTL8*, and *LOC101928978* were significantly correlated with BRCA patients’ overall survival (OS) from the training set. Figure 2A illustrated the hazard ratio (HR) and significant *P* value of those genes. LASSO Cox-regression analysis determined six genes based on which the partial likelihood deviance (PLD) had the lowest value (Figure 2B), and the regression model was built as an equation: risk score = 0.0137*mRNA level of *HOXB13* + 0.0928*mRNA level of *NKX2-2* + 0.0343*mRNA level of *ADAMTS20* + 0.103*mRNA level of *LOC284930* + 0.0419*mRNA level of *ACTL8* + 0.0871*mRNA level of *LOC101928978*.

### Risk score is an independent marker for radiotherapy sensitivity prediction

**Figure 3:**
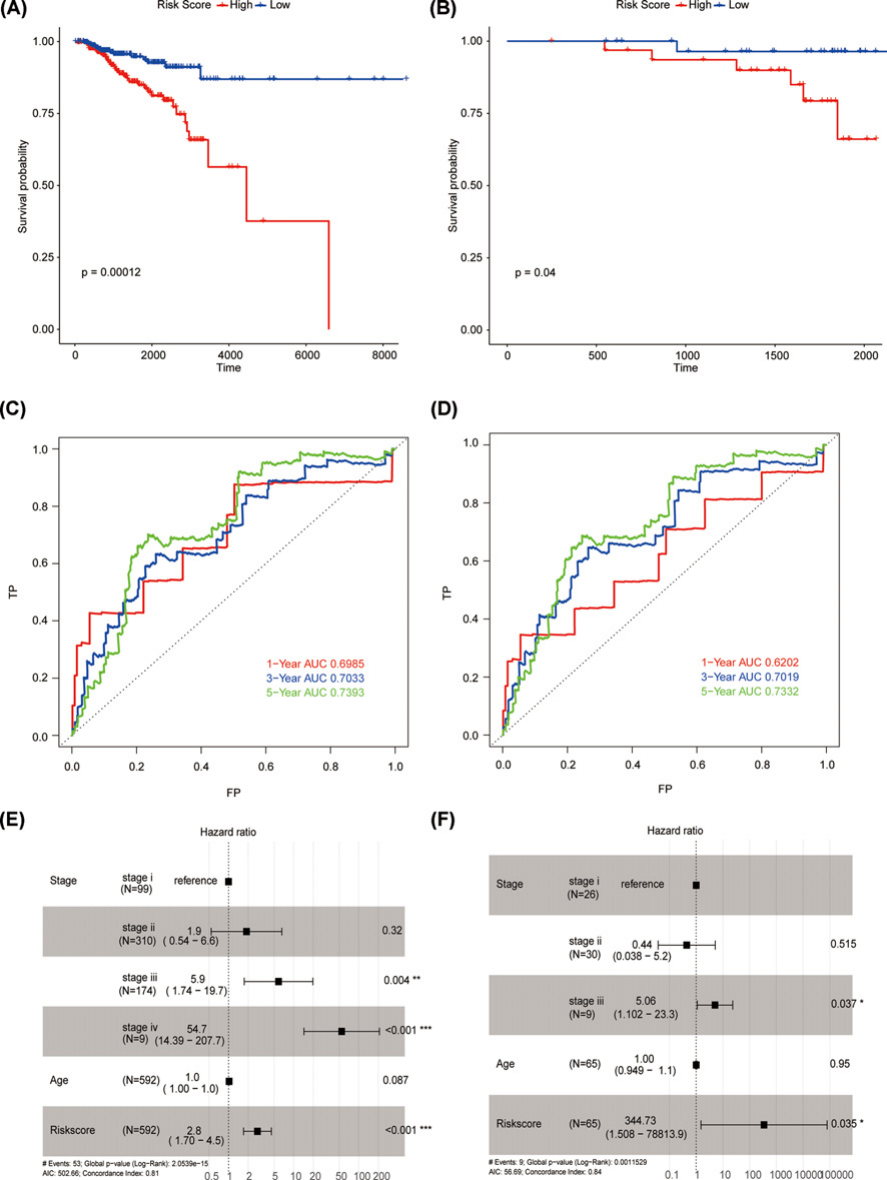
Risk score is an independent factor for BRCA radiotherapy sensitivity (**A**) Kaplan–Meier (KM) plot of BRCA patients in radiotherapy group from TCGA stratified by the median risk score. P value calculated using log-rank test was provided. (**B**) KM plot of BRCA patients in radiotherapy group from ICGC stratified by the median risk score. *P* value calculated using log-rank test was provided. (**C**) ROC curve for the prediction model in the TCGA training set (C). (**D**) ROC curve for the prediction model in the ICGC validation set. (**E**) Forest plot of multivariate Cox regression analysis of the training set indicated the risk score as an independent marker for BRCA radiotherapy sensitivity. (**F**) Forest plot of multivariate Cox regression analysis of the testing set indicated the risk score as an independent marker for BRCA radiotherapy sensitivity.

Samples that have been treated with radiation from both training and testing set were assigned scores according to the risk score equation. Kaplan–Meier (KM) survival analysis stratified by the median risk score uncovered its unfavorable survival role for BRCA patients in training set (Figure 3A), which should indicate that high risk score correlated with insensitive response to radiotherapy. The details of training set, including risk score, age, stage, OS and status, were shown in Supplementary Table S1. Besides, there were 65 patients had radiotherapy information out of the 1019 breast cancer samples from ICGC, which were also assigned risk scores according to the risk score equation. Kaplan–Meier analysis indicated significantly inferior OS of samples with higher risk score. To further explore if risk score was independent of other common clinicopathological features in predicting radiotherapy sensitivity, we performed multivariate Cox regression analysis by simultaneously taking age, stage, and risk score into account. The difference of age and stage between the high- and low-risk groups in the TCGA training set and the ICGC validation set was shown in Supplementary Figure S1. The ROC curve showed that prediction model was able to predict the prognosis reliably (Figure 3C,E). As a result, risk score was proved to significantly correlate with the overall survival of BRCA patients that have treated with radiation in both TCGA (Figure 3E) and ICGC sets (Figure 3F).

### Nomogram could robustly estimate OS probability

**Figure 4:**
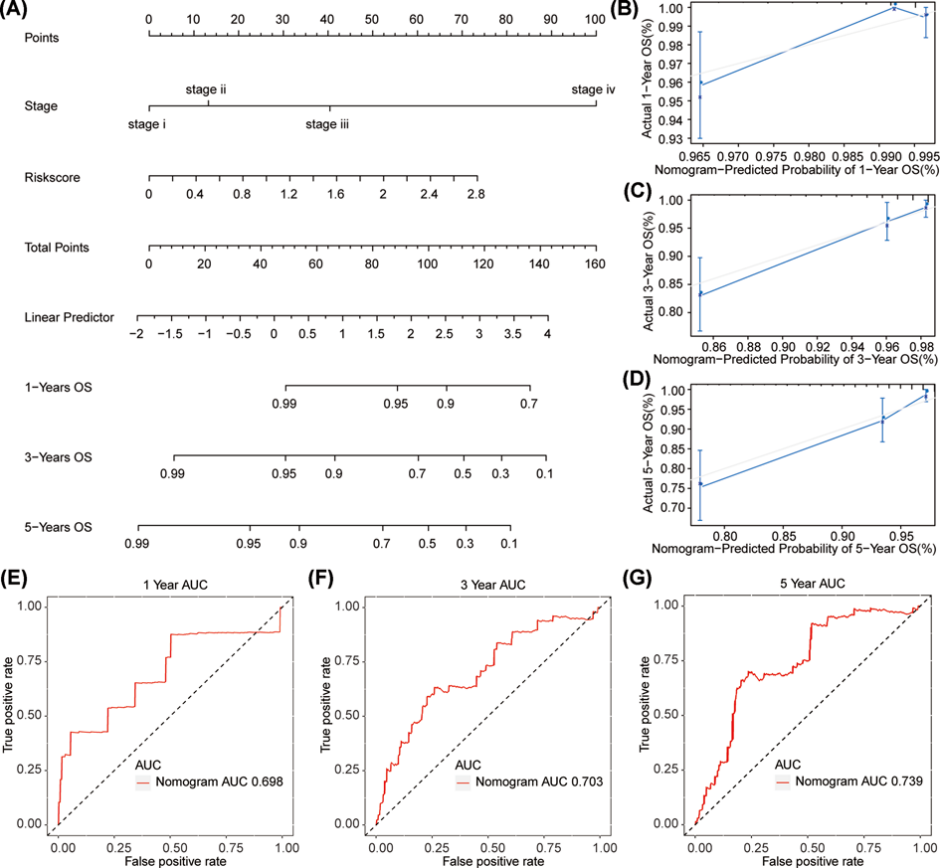
Nomogram for BRCA OS probability estimation after radiotherapy (**A**) Nomogram combing TNM stage and risk score. A single point is assigned to the stage or risk score that is perpendicular to the point line. Total point is assigned to every BRCA sample by combining the sample's risk score and TNM stage corresponded point. OS probability was estimated according to the corresponding total point. Calibration curve to evaluate the ability of the nomogram in estimating 1-year (**B**), 3-year (**C**), and 5-year (**D**) OS probability. ROC curve for the nomogram in estimating 1-year (**E**), 3-year (**F**), and 5-year (**G**).

By incorporating those independent prognostic signatures in radiation-treated BRCA patients, i.e. TNM stage and risk score, we constructed a nomogram model as shown in Figure 4A. Calibration curve indicated that the deviation is very small between the actual and nomogram estimated 1-year (Figure 4B), 3-year (Figure 4C), and 5-year (Figure 4D) OS probability for BRCA patients after radiotherapy. The area under the ROC curve (AUC) of the nomogram estimated 1-, 3-, and 5-year model was 0.698, 0.703, and 0.739, respectively (Figure 4E–G). This illustrated the potential of the nomogram in clinical directing after radiotherapy.

### Risk score is a signature for immune response of BRCA patients

**Figure 5:**
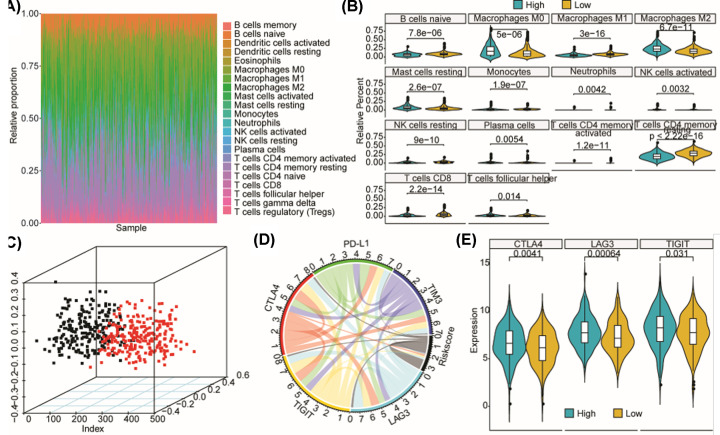
The radiotherapy sensitivity signature is related to immune response in BRCA patients (**A**) Landscape of relative proportions of 22 TIICs across BRCA samples in TCGA dataset. (**B**) Violin plots illustrating the relative proportions of the 14 TIICs exhibiting significantly different infiltrating degree between BRCA samples with high- and low-risk score. (**C**) PCA plot of BRCA samples based on the relative proportions of the 14 significantly different TIICs. (**D**) Chord diagram illustrating the Spearman correlations between mRNA levels of five key immune checkpoint receptors, as well as between the mRNA levels of five key immune checkpoint receptors and the risk score. The broader the line between two mRNAs or between risk score and mRNAs, the greater the correlation is. (**E**) Violin plots showing mRNA levels of CTLA4, LAG3, and TIGIT in BRCA samples stratified by the median risk score.

Immunotherapy is becoming a pivotal treatment method for multiple cancers and those targeting immune checkpoint receptors, including PD-L1, CTLA4, TIGIT, LAG3, and TIM3, represent the most promising ones. In addition, TIICs also deeply affect the immune response in tumors. Here, we investigated the landscape of relative proportions of the 22 TIICs across all the BRCA tumor samples in the TCGA dataset (Figure 5A). There are 14 TIICs that exhibit significantly different relative proportions between BRCA tumor samples with high- and low-risk scores stratified by the median risk score as shown in Figure 5B. What's more, principle component analysis (PCA) based on those 14 TIICs could definitely sperate BRCA tumor samples with high- and low-risk scores (Figure 5C). Those results partially illustrated the potential of the radiotherapy sensitivity signature in immune response estimation.

We further explored the associations between risk score and mRNA levels of five key immune checkpoint receptors, including PD-L1, CTLA4, TIGIT, LAG3, and TIM3. As a result, the mRNA levels were significantly positively correlated with each other among the five immune checkpoint receptors, while the risk score is negatively correlated with the mRNA levels of those immune checkpoint receptors (Figure 5D). Spearman correlations among those mRNA levels and risk scores were provided in Supplementary Table S2. In addition, there are three checkpoint receptors, i.e. CTLA4, LAG3, and TIGIT, exhibited significantly higher mRNA levels in BRCA samples with high-risk score than those with low-risk score as shown in Figure 5E. Those should illustrate the potential feasibility of the signature used as an immunotherapy sensitivity marker for BRCA in addition to its radiotherapy sensitivity estimation role.

## Discussion

Deep learning models have been widely used in the area of bioinformatics, including biomedical signal processing, biomedical imaging, omics, and tumor classification [16,17]. Some deep neural network models are capable of learning a meaningful latent space, which could be used to explore and generate hypothetical gene expression profiles under various types of molecular and genetic perturbation, or to predict a tumor’s response to specific therapies [18]. In this study, we identified 603 genes that differently expressed in BRCA tumor samples compared with paracancerous samples. Among them, the most representative genes were screened using a neural network-based learning technique, which is mainly used for data dimensionality reduction or feature extraction. Fifty genes were considered relatively important, and their associations with BRCA patients’ OS were further investigated by univariate Cox-regression analysis. Finally, we reduce the number of candidate key genes to 6 by using LASSO Cox-regression analysis, including *HOXB13, NKX2-2, ADAMTS20, LOC284930, ACTL8*, and *LOC101928978*.

Homeobox B13 (*HOXB13*) is a member of the human ANTP class homeobox gene and is located at 17q21.32 of the chromosome [19]. The expression of *HOXB13* has been associated with the development of several cancers. For example, *HOXB13* was reported to be able to mediate NF-κB/p65 pathway and regulate the proliferation and metastasis of esophageal squamous cell carcinoma [20]. Besides, *HOXB13* expression was significantly associated with prostate ductal type adenocarcinoma and biochemical recurrence (BCR) as well as shorter BCR-free survival [21]. In BRCA, *HOXB13* has long been identified as a prognostic biomarker [21,22]. *HOXB13* can confer tamoxifen resistance by directly downregulating the transcription of estrogen receptor α (ERα), and transcriptionally up-regulated interleukin (IL)-6, activating the mTOR pathway via STAT3 phosphorylation to promote the proliferation of BRCA tumor cells and the recruitment of fibroblast, leading to disease progression and recurrence [23].

A disintegrin and metalloprotease domains with thrombospondins motifs (ADAMTSs) are complex extracellular proteases that have been related to both oncogenic and tumor-protective functions [24]. Multiple subtypes of ADAMTSs was proved to play a role in the development of BRCA. For example, *ADAMTS1* expression was decreased in BRCA, which can stimulate the migration and invasion of breast cancer cells *in vitro*. It can also respond to VEGF, and implicate in tissue remodeling events observed in cancer development [25,26]. *ADAMTS6* suppressed tumor progression via the ERK signaling pathway and might serve as a prognostic marker in BRCA [27]. The expression level of *ADAMTS20* was also significantly associated with the histological grade of breast invasive ductal carcinoma [28]. Meanwhile, the actin-like protein 8 (ACTL8) protein was reported to be highly expressed in BRCA specimens and is closely correlated with the clinicopathological features and prognosis [30].

*NKX2-2* is an oligodendroglial and astrocytic lineage marker, and also a useful immunohistochemical marker for Ewing sarcoma [29,30]. Yang et al. indicated that the high expression of *NKX2-2* was significantly correlated with the poor OS for all invasive breast cancer patients [31]. *NKX2-2* is one of the downstream target genes of GLI1 in the Sonic hedgehog (Shh) signaling pathway, and impairment of this pathway can result in both birth defects and cancer [32]. However, the role of *NKX2-2* in breast cancer has not been revealed. Unfortunately, little research has been done on the functions of *LOC284930* and *LOC101928978*. Only one report suggested that the expression of *LOC284930* was positively correlated with ERG overexpression, which is the most frequent genomic rearrangement in prostate cancer [33].

Overall, 3 of the 6 key genes have been confirmed to be involved in the development of BRCA and are expected to serve as prognostic indicators. To our knowledge, this is the first time these genes were linked to the radiotherapy sensitivity of BRCA.

## Conclusion

In the present study, a signature for BRCA radiotherapy sensitivity prediction was developed based on the expression of six characteristic DEGs, including *HOXB13, NKX2-2, ADAMTS20, LOC284930, ACTL8* and *LOC101928978*, in BRCA tumor samples compared with their paracancerous samples for the first time. A radiotherapy sensitivity prediction signature was constructed with these characteristic DEGs, and this signature was proved to be reliable. In addition, this signature exhibited the potential as an immune-response signature in BRCA.

## Supplementary Material

Supplementary Figure S1 and Tables S1-S2

## Data Availability

Data were obtained from TCGA (http://tcgaportal.org/), and ICGC (https://icgc.org/).

## Abbreviations

ACTL8: actin-like protein 8
BRCA: breast cancer
DEG: differential expression gene
ICGC: International Cancer Genome Consortium
IL: interleukin
TCGA: the Cancer Genome Atlas
TIIC: tumor infiltrating immune cell
