# Supplementary material for: A six-gene-based signature for breast cancer radiotherapy sensitivity estimation
Source: Biosci Rep. 2020 Dec 2;40(12):BSR20202376. doi: 10.1042/BSR20202376 (PMC7711058; doi:10.1042/BSR20202376)
Supplement: Supplementary Figure S1 and Tables S1-S2 [file BSR-2020-2376_supp.pdf]

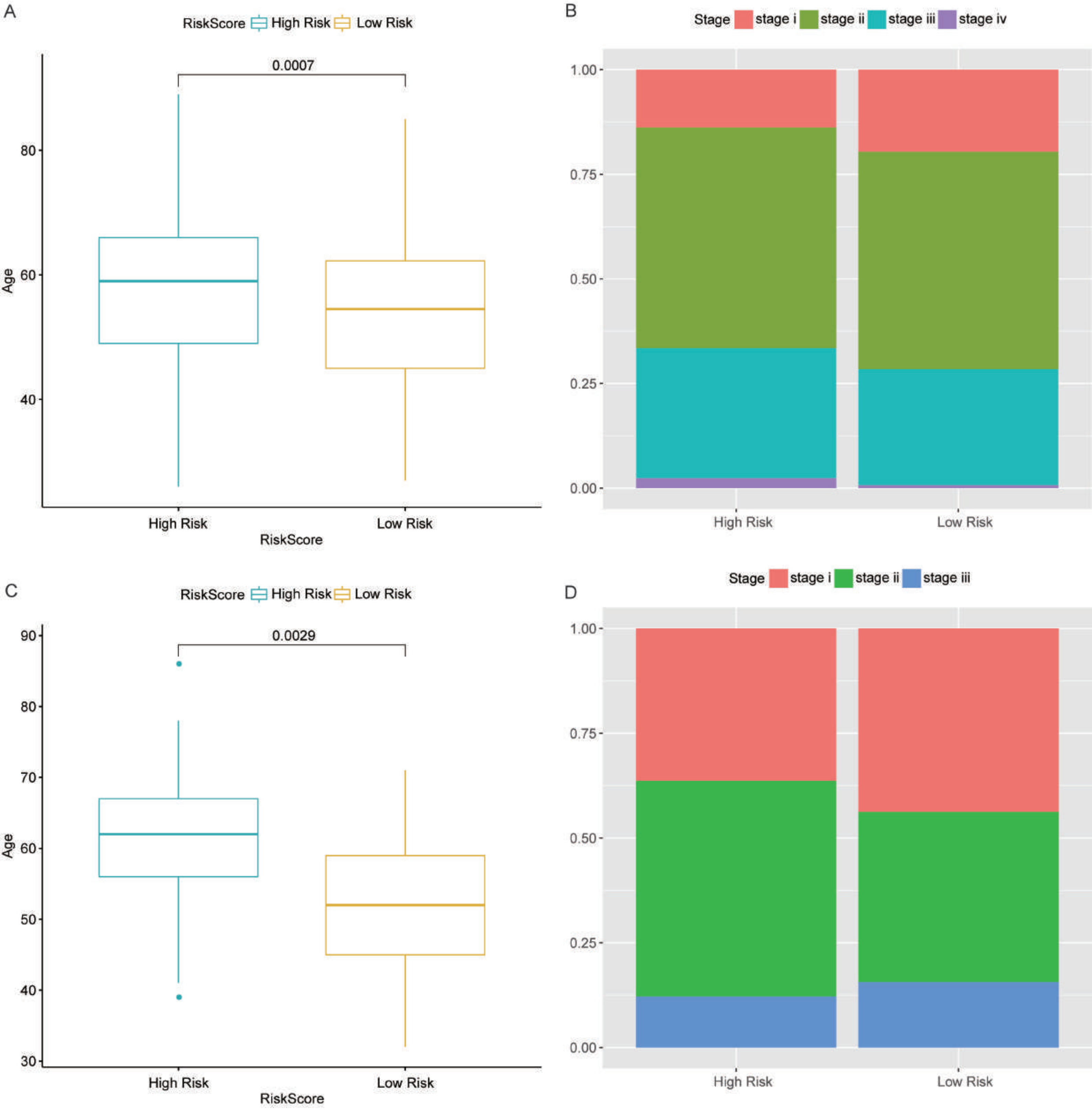
A
RiskScore High Risk Low Risk
0.0007
Age
80
60
40
High Risk
Low Risk
RiskScore
B
Stage stage i stage ii stage iii stage iv
1.00
0.75
0.50
0.25
0.00
High Risk
Low Risk
C
RiskScore High Risk Low Risk
0.0029
Age
90
80
70
60
50
40
30
High Risk
Low Risk
RiskScore
D
Stage stage i stage ii stage iii
1.00
0.75
0.50
0.25
0.00
High Risk
Low Risk

Supplementary Figure S1. The differences of age and stage between the high- and low-risk groups.

Table S1 The detail information of training set.

id	RiskScore	Age	Stage	os	status
TCGA-BH-A0B5	Low Risk	40	stage iii	2136	0
TCGA-BH-A0B3	Low Risk	53	stage ii	1203	0
TCGA-A7-A3IY	Low Risk	71	stage i	345	0
TCGA-A2-A0YC	Low Risk	59	stage ii	990	0
TCGA-BH-A0HW	Low Risk	62	stage i	1561	0
TCGA-E2-A15M	Low Risk	66	stage ii	336	1
TCGA-E9-A1NA	Low Risk	58	stage ii	1112	0
TCGA-BH-A0E0	Low Risk	38	stage iii	134	0
TCGA-A2-A0EM	Low Risk	73	stage i	3094	0
TCGA-E9-A1RI	Low Risk	43	stage iii	1449	0
TCGA-BH-A0DH	Low Risk	63	stage ii	1156	0
TCGA-GM-A2DM	Low Risk	57	stage ii	3226	0
TCGA-BH-A0DG	Low Risk	30	stage ii	2041	0
TCGA-BH-A0DL	Low Risk	64	stage ii	2381	0
TCGA-B6-A0RV	Low Risk	42	stage iii	5156	0
TCGA-A7-A13F	Low Risk	44	stage iii	765	0
TCGA-E2-A1L7	Low Risk	40	stage iii	1836	0
TCGA-S3-AA15	Low Risk	51	stage ii	525	0
TCGA-E9-A1N6	Low Risk	52	stage ii	678	1
TCGA-A2-A04R	Low Risk	36	stage i	3709	0
TCGA-A8-A07S	Low Risk	73	stage ii	243	0
TCGA-A2-A0EO	Low Risk	54	stage i	2442	0
TCGA-A7-A3IZ	Low Risk	62	stage ii	322	0
TCGA-BH-A0H9	Low Risk	69	stage ii	1247	0
TCGA-E2-A156	Low Risk	61	stage i	726	0
TCGA-GI-A2C8	Low Risk	63	stage iii	225	0
TCGA-BH-A0W3	Low Risk	58	stage ii	728	0

TCGA-A7-A0DC	Low Risk	63	stage i	906	0
TCGA-A7-A0CD	Low Risk	66	stage i	1165	0
TCGA-D8-A27E	Low Risk	66	stage i	530	0
TCGA-E9-A1RH	Low Risk	63	stage ii	1417	0
TCGA-A7-A13F	Low Risk	44	stage iii	765	0
TCGA-BH-A0B8	Low Risk	64	stage i	1569	0
TCGA-E9-A249	Low Risk	45	stage ii	217	0
TCGA-E2-A1L8	Low Risk	52	stage ii	2240	0
TCGA-EW-A1P4	Low Risk	43	stage ii	907	0
TCGA-AO-A03R	Low Risk	57	stage ii	2091	0
TCGA-BH-A0E7	Low Risk	79	stage ii	1363	0
TCGA-BH-A0B7	Low Risk	42	stage ii	2559	0
TCGA-BH-A0B4	Low Risk	65	stage iii	1191	0
TCGA-AR-A2LJ	Low Risk	40	stage iii	2632	0
TCGA-BH-A0BZ	Low Risk	59	stage iii	2255	0
TCGA-A7-A0DB	Low Risk	56	stage ii	1007	0
TCGA-D8-A1XM	Low Risk	57	stage i	538	0
TCGA-BH-A0B5	Low Risk	40	stage iii	2136	0
TCGA-AO-A0JM	Low Risk	40	stage ii	2184	0
TCGA-GM-A2DC	Low Risk	57	stage ii	2535	0
TCGA-BH-A0DP	Low Risk	60	stage ii	476	0
TCGA-E2-A153	Low Risk	51	stage ii	707	0
TCGA-BH-A0BO	Low Risk	54	stage i	2197	0
TCGA-D8-A1JI	Low Risk	54	stage ii	577	0
TCGA-AC-A3TN	Low Risk	75	stage ii	456	0
TCGA-BH-A0DT	Low Risk	41	stage ii	2403	0
TCGA-WT-AB44	Low Risk	77	stage i	883	0
TCGA-A2-A0D2	Low Risk	45	stage ii	1027	0
TCGA-E2-A572	Low Risk	72	stage iii	1208	0

TCGA-BH-A0EI	Low Risk	51	stage ii	1926	0
TCGA-E9-A1R7	Low Risk	64	stage ii	1467	0
TCGA-BH-A0H6	Low Risk	82	stage i	747	0
TCGA-A2-A0CS	Low Risk	73	stage iv	2348	1
TCGA-LD-A66U	Low Risk	44	stage ii	646	0
TCGA-5T-A9QA	Low Risk	52	stage ii	303	0
TCGA-A2-A1G6	Low Risk	50	stage iii	501	0
TCGA-AO-A0J9	Low Risk	61	stage iii	1613	0
TCGA-B6-A0I2	Low Risk	45	stage i	4361	0
TCGA-BH-A0BS	Low Risk	55	stage iii	2612	0
TCGA-EW-A1OX	Low Risk	43	stage ii	911	0
TCGA-BH-A0C3	Low Risk	47	stage i	2709	0
TCGA-LL-A7SZ	Low Risk	49	stage ii	594	0
TCGA-E2-A1LH	Low Risk	59	stage i	3247	0
TCGA-MS-A51U	Low Risk	44	stage ii	681	0
TCGA-BH-A6R8	Low Risk	46	stage ii	293	0
TCGA-AO-A1KT	Low Risk	78	stage ii	541	0
TCGA-E2-A1IG	Low Risk	45	stage ii	2140	0
TCGA-BH-A0DV	Low Risk	54	stage iii	2064	0
TCGA-EW-A1P0	Low Risk	55	stage ii	1251	0
TCGA-OL-A5RU	Low Risk	63	stage ii	1219	0
TCGA-A2-A3KD	Low Risk	47	stage iii	1206	0
TCGA-E2-A1B1	Low Risk	45	stage ii	2653	0
TCGA-E2-A107	Low Risk	54	stage iii	1047	0
TCGA-AR-A0TZ	Low Risk	43	stage iii	3262	1
TCGA-B6-A1KI	Low Risk	63	stage i	2236	0
TCGA-A7-A0CE	Low Risk	57	stage ii	1074	0
TCGA-AR-A24O	Low Risk	43	stage iii	3607	0
TCGA-E2-A14N	Low Risk	37	stage ii	1434	0

TCGA-BH-A0BT	Low Risk	56	stage ii	2365	0
TCGA-E2-A1II	Low Risk	51	stage i	1025	0
TCGA-BH-A0BJ	Low Risk	41	stage ii	660	0
TCGA-A7-A5ZW	Low Risk	47	stage ii	326	0
TCGA-E9-A1N4	Low Risk	41	stage iii	1000	0
TCGA-BH-A0BA	Low Risk	51	stage iii	1132	0
TCGA-E9-A24A	Low Risk	69	stage ii	747	0
TCGA-BH-A0DQ	Low Risk	42	stage ii	98	0
TCGA-BH-A0BT	Low Risk	56	stage ii	2365	0
TCGA-A2-A3XT	Low Risk	45	stage ii	2770	0
TCGA-A2-A0ER	Low Risk	63	stage i	2263	0
TCGA-D8-A27F	Low Risk	40	stage ii	488	0
TCGA-S3-A6ZG	Low Risk	71	stage ii	562	0
TCGA-AO-A0J7	Low Risk	71	stage ii	618	0
TCGA-E2-A15K	Low Risk	58	stage ii	275	0
TCGA-B6-A0WZ	Low Risk	50	stage ii	6292	0
TCGA-BH-A0C3	Low Risk	47	stage i	2709	0
TCGA-BH-A0AU	Low Risk	45	stage ii	1914	0
TCGA-BH-A0W7	Low Risk	49	stage i	1363	0
TCGA-E9-A3X8	Low Risk	48	stage ii	926	0
TCGA-E2-A1IO	Low Risk	37	stage i	1855	0
TCGA-A7-A0CE	Low Risk	57	stage ii	1074	0
TCGA-E9-A1N4	Low Risk	41	stage iii	1000	0
TCGA-GM-A4E0	Low Risk	67	stage iii	2191	0
TCGA-A8-A09R	Low Risk	82	stage ii	273	0
TCGA-A8-A095	Low Risk	45	stage i	1277	0
TCGA-AR-A254	Low Risk	50	stage iii	2605	0
TCGA-EW-A2FW	Low Risk	52	stage ii	672	0
TCGA-AO-A0J8	Low Risk	61	stage ii	680	0

TCGA-AR-A24R	Low Risk	45	stage iii	3430	0
TCGA-BH-A0B0	Low Risk	56	stage i	2477	0
TCGA-B6-A0IQ	Low Risk	40	stage iii	4285	0
TCGA-D8-A73W	Low Risk	79	stage iii	385	1
TCGA-A8-A0A9	Low Risk	80	stage ii	822	0
TCGA-GI-A2C9	Low Risk	58	stage ii	3342	0
TCGA-E9-A22H	Low Risk	42	stage ii	1232	0
TCGA-A2-A25E	Low Risk	34	stage iii	3204	0
TCGA-AR-A24M	Low Risk	38	stage iii	3660	0
TCGA-BH-A0EB	Low Risk	69	stage i	745	0
TCGA-EW-A1OW	Low Risk	58	stage ii	694	0
TCGA-A2-A4S2	Low Risk	62	stage iii	643	0
TCGA-GM-A3XN	Low Risk	44	stage iii	2019	0
TCGA-BH-A18G	Low Risk	81	stage i	149	0
TCGA-S3-AA11	Low Risk	67	stage ii	421	0
TCGA-A2-A0CP	Low Risk	60	stage i	2813	0
TCGA-B6-A408	Low Risk	55	stage iii	2072	0
TCGA-EW-A1PA	Low Risk	59	stage ii	575	0
TCGA-E2-A1IJ	Low Risk	57	stage i	865	0
TCGA-E2-A1LB	Low Risk	41	stage ii	2306	0
TCGA-BH-A0BJ	Low Risk	41	stage ii	660	0
TCGA-AQ-A1H3	Low Risk	49	stage iii	989	0
TCGA-BH-A0DE	Low Risk	62	stage ii	2372	0
TCGA-A2-A25B	Low Risk	39	stage ii	1291	0
TCGA-EW-A2FV	Low Risk	39	stage iii	788	0
TCGA-B6-A402	Low Risk	47	stage i	2281	0
TCGA-EW-A1P5	Low Risk	77	stage ii	703	0
TCGA-B6-A0RU	Low Risk	40	stage i	8605	0
TCGA-GM-A2DO	Low Risk	54	stage i	2596	0

TCGA-E2-A153	Low Risk	51	stage ii	707	0
TCGA-BH-A5IZ	Low Risk	51	stage ii	567	0
TCGA-B6-A400	Low Risk	43	stage iii	215	0
TCGA-A8-A093	Low Risk	61	stage ii	546	0
TCGA-AC-A2FF	Low Risk	40	stage ii	2759	0
TCGA-BH-A0BA	Low Risk	51	stage iii	1132	0
TCGA-BH-A0HI	Low Risk	78	stage i	620	0
TCGA-AR-A1AN	Low Risk	46	stage ii	2920	0
TCGA-AC-A2FK	Low Risk	45	stage iii	2650	0
TCGA-A2-A3XW	Low Risk	42	stage ii	1712	0
TCGA-A2-A0T3	Low Risk	37	stage i	1516	0
TCGA-B6-A0RN	Low Risk	60	stage i	8008	0
TCGA-S3-AA14	Low Risk	47	stage i	529	0
TCGA-E2-A1B4	Low Risk	74	stage iii	1004	1
TCGA-A2-A04N	Low Risk	66	stage i	4354	0
TCGA-A7-A3J1	Low Risk	63	stage i	343	0
TCGA-D8-A1X8	Low Risk	62	stage iii	783	0
TCGA-PE-A5DE	Low Risk	41	stage ii	2645	0
TCGA-AO-A12F	Low Risk	36	stage ii	1842	0
TCGA-BH-A0DP	Low Risk	60	stage ii	476	0
TCGA-AC-A62Y	Low Risk	79	stage ii	530	0
TCGA-A7-A4SB	Low Risk	56	stage iii	418	0
TCGA-V7-A7HQ	Low Risk	75	stage iii	2033	0
TCGA-A8-A09V	Low Risk	51	stage ii	457	0
TCGA-A8-A07C	Low Risk	57	stage ii	1034	0
TCGA-E2-A56Z	Low Risk	69	stage ii	252	0
TCGA-AO-A0JG	Low Risk	49	stage iii	798	0
TCGA-A2-A0T6	Low Risk	50	stage ii	575	0
TCGA-A2-A0YE	Low Risk	48	stage ii	554	0

TCGA-A8-A08I	Low Risk	53	stage ii	365	0
TCGA-AO-A0J4	Low Risk	41	stage i	1587	0
TCGA-D8-A1XA	Low Risk	64	stage i	839	0
TCGA-AR-A2LE	Low Risk	69	stage i	5062	0
TCGA-E9-A1R6	Low Risk	63	stage ii	339	0
TCGA-AR-A0TP	Low Risk	43	stage ii	4275	0
TCGA-AO-A129	Low Risk	29	stage ii	3286	0
TCGA-AR-A1AY	Low Risk	65	stage i	1026	0
TCGA-A2-A0EW	Low Risk	53	stage iii	1884	1
TCGA-D8-A1XS	Low Risk	48	stage iii	496	0
TCGA-BH-A201	Low Risk	64	stage i	856	0
TCGA-EW-A1P1	Low Risk	68	stage iii	1210	0
TCGA-A2-A0T1	Low Risk	55	stage iii	521	0
TCGA-AR-A1AQ	Low Risk	49	stage ii	3021	0
TCGA-E2-A15P	Low Risk	61	stage i	595	0
TCGA-BH-A0DH	Low Risk	63	stage ii	1156	0
TCGA-BH-A0BC	Low Risk	60	stage iii	974	0
TCGA-E2-A15G	Low Risk	76	stage ii	554	0
TCGA-AR-A2LO	Low Risk	46	stage ii	1198	0
TCGA-GM-A3XG	Low Risk	46	stage iii	1330	0
TCGA-A2-A0SX	Low Risk	48	stage i	1534	0
TCGA-A7-A6VY	Low Risk	48	stage ii	266	0
TCGA-D8-A1JB	Low Risk	54	stage ii	1688	0
TCGA-A8-A086	Low Risk	59	stage ii	396	0
TCGA-A8-A09T	Low Risk	68	stage ii	579	0
TCGA-OL-A6VO	Low Risk	43	stage i	858	0
TCGA-BH-A0E0	Low Risk	38	stage iii	134	0
TCGA-B6-A2IU	Low Risk	62	stage ii	5176	0
TCGA-A2-A0ET	Low Risk	58	stage iii	1066	0

TCGA-GM-A2DL	Low Risk	50	stage i	3519	0
TCGA-A7-A5ZV	Low Risk	62	stage ii	368	0
TCGA-E2-A1IG	Low Risk	45	stage ii	2140	0
TCGA-B6-A0RE	Low Risk	61	stage ii	7777	0
TCGA-E2-A10A	Low Risk	41	stage ii	1229	0
TCGA-B6-A0RI	Low Risk	44	stage iii	7126	0
TCGA-E2-A1L9	Low Risk	40	stage ii	598	0
TCGA-A8-A07U	Low Risk	66	stage iii	760	0
TCGA-AC-A3QP	Low Risk	79	stage ii	675	0
TCGA-AR-A0U0	Low Risk	73	stage ii	1988	0
TCGA-A2-A0CK	Low Risk	60	stage iii	4159	0
TCGA-BH-A0H0	Low Risk	69	stage i	461	0
TCGA-S3-A6ZH	Low Risk	29	stage iii	641	0
TCGA-EW-A1J1	Low Risk	38	stage ii	575	0
TCGA-AR-A24V	Low Risk	52	stage ii	3203	0
TCGA-BH-A28O	Low Risk	50	stage iii	1120	0
TCGA-A2-A0YL	Low Risk	48	stage iii	1474	0
TCGA-E9-A5UP	Low Risk	63	stage ii	803	0
TCGA-AN-A0G0	Low Risk	56	stage ii	16	0
TCGA-AC-A2QJ	Low Risk	48	stage iii	446	1
TCGA-A7-A6VW	Low Risk	48	stage ii	285	0
TCGA-BH-A0DS	Low Risk	71	stage iii	78	0
TCGA-AR-A1AR	Low Risk	50	stage iii	524	1
TCGA-C8-A12Y	Low Risk	44	stage ii	1476	0
TCGA-E9-A1NE	Low Risk	28	stage ii	1088	0
TCGA-BH-A0H7	Low Risk	65	stage iii	702	0
TCGA-B6-A40C	Low Risk	51	stage ii	2164	0
TCGA-E9-A1RI	Low Risk	43	stage iii	1449	0
TCGA-AR-A0TQ	Low Risk	27	stage iii	2991	0

TCGA-LL-A8F5	Low Risk	61	stage ii	596	0
TCGA-E2-A14R	Low Risk	62	stage ii	1174	0
TCGA-D8-A1JD	Low Risk	41	stage ii	552	0
TCGA-A8-A08C	Low Risk	65	stage ii	881	0
TCGA-E9-A6HE	Low Risk	45	stage iii	847	0
TCGA-A2-A0ST	Low Risk	62	stage ii	3017	0
TCGA-E2-A108	Low Risk	64	stage iii	837	0
TCGA-GM-A3XL	Low Risk	49	stage ii	2108	0
TCGA-BH-A0AV	Low Risk	52	stage i	1820	0
TCGA-BH-A0W5	Low Risk	77	stage ii	1288	0
TCGA-E2-A576	Low Risk	69	stage i	1043	0
TCGA-GI-A2C8	Low Risk	63	stage iii	225	0
TCGA-D8-A1XL	Low Risk	34	stage ii	606	0
TCGA-BH-A0C0	Low Risk	62	stage ii	1270	0
TCGA-A2-A0CQ	Low Risk	62	stage i	2695	0
TCGA-A7-A3J0	Low Risk	62	stage ii	313	0
TCGA-BH-A0E9	Low Risk	53	stage ii	2489	0
TCGA-A8-A07L	Low Risk	58	stage iii	975	0
TCGA-E2-A150	Low Risk	48	stage ii	1935	0
TCGA-BH-A0B3	Low Risk	53	stage ii	1203	0
TCGA-BH-A0DV	Low Risk	54	stage iii	2064	0
TCGA-A8-A07P	Low Risk	68	stage ii	334	0
TCGA-BH-A0C0	Low Risk	62	stage ii	1270	0
TCGA-D8-A27T	Low Risk	53	stage iii	398	0
TCGA-AO-A0JJ	Low Risk	54	stage ii	1887	0
TCGA-A2-A1FX	Low Risk	61	stage iii	1847	0
TCGA-BH-A0GZ	Low Risk	62	stage ii	328	0
TCGA-E2-A1LH	Low Risk	59	stage i	3247	0
TCGA-OL-A66N	Low Risk	59	stage iii	792	0

TCGA-A8-A09M	Low Risk	75	stage iii	1006	0
TCGA-A2-A04P	Low Risk	36	stage iii	548	1
TCGA-D8-A1JH	Low Risk	56	stage i	426	0
TCGA-BH-A0H3	Low Risk	46	stage i	1928	0
TCGA-B6-A0X7	Low Risk	62	stage iii	1781	1
TCGA-E2-A15C	Low Risk	61	stage i	694	0
TCGA-OL-A66O	Low Risk	39	stage ii	528	0
TCGA-OL-A5RY	Low Risk	52	stage ii	752	0
TCGA-AO-A0JF	Low Risk	68	stage ii	1980	0
TCGA-A2-A3XU	Low Risk	35	stage ii	912	1
TCGA-AR-A2LH	Low Risk	55	stage iii	616	1
TCGA-A2-A25C	Low Risk	50	stage ii	523	0
TCGA-A8-A091	Low Risk	61	stage ii	1004	0
TCGA-EW-A1PE	Low Risk	56	stage ii	320	0
TCGA-A2-A0CO	Low Risk	85	stage ii	1468	1
TCGA-A8-A07I	Low Risk	69	stage iii	426	0
TCGA-A2-A0EU	Low Risk	79	stage i	1043	0
TCGA-A2-A1G0	Low Risk	49	stage ii	616	0
TCGA-AC-A6IV	Low Risk	47	stage ii	568	0
TCGA-BH-A0HQ	Low Risk	56	stage ii	1121	0
TCGA-AR-A1AW	Low Risk	65	stage ii	2632	0
TCGA-A7-A425	Low Risk	70	stage iii	447	0
TCGA-BH-A0AW	Low Risk	56	stage ii	622	0
TCGA-GM-A2DK	Low Risk	58	stage i	2645	0
TCGA-E2-A1AZ	Low Risk	63	stage ii	2329	0
TCGA-E9-A5FK	Low Risk	60	stage iii	812	0
TCGA-AR-A24K	Low Risk	46	stage ii	1548	0
TCGA-AO-A0JB	Low Risk	50	stage iii	1542	0
TCGA-E9-A229	Low Risk	37	stage i	1148	0

TCGA-D8-A1XB	Low Risk	62	stage ii	552	0
TCGA-AQ-A1H2	Low Risk	84	stage iii	475	0
TCGA-AO-A12E	Low Risk	51	stage ii	2142	0
TCGA-LL-A73Z	Low Risk	55	stage iv	227	1
TCGA-AR-A0U3	Low Risk	59	stage ii	4080	0
TCGA-E2-A15D	Low Risk	47	stage ii	526	0
TCGA-EW-A424	Low Risk	51	stage iii	715	0
TCGA-EW-A6SC	Low Risk	60	stage ii	952	0
TCGA-A8-A07J	High Risk	35	stage ii	365	0
TCGA-EW-A1IZ	High Risk	53	stage iii	554	0
TCGA-AO-A0JC	High Risk	64	stage ii	1547	0
TCGA-BH-A0BW	High Risk	71	stage i	2371	0
TCGA-A8-A099	High Risk	76	stage i	304	0
TCGA-AC-A2FF	High Risk	40	stage ii	2759	0
TCGA-AC-A3BB	High Risk	46	stage iii	987	0
TCGA-BH-A0DT	High Risk	41	stage ii	2403	0
TCGA-A2-A0CV	High Risk	41	stage ii	3011	0
TCGA-AC-A62V	High Risk	58	stage iv	348	1
TCGA-A8-A09A	High Risk	40	stage ii	304	0
TCGA-BH-A0HB	High Risk	55	stage i	806	0
TCGA-A8-A079	High Risk	69	stage iii	274	0
TCGA-AC-A2FE	High Risk	62	stage iii	2636	1
TCGA-E2-A15E	High Risk	40	stage ii	630	0
TCGA-OL-A5D7	High Risk	70	stage ii	1780	0
TCGA-E2-A1B5	High Risk	46	stage ii	984	0
TCGA-E2-A1L7	High Risk	40	stage iii	1836	0
TCGA-B6-A0IH	High Risk	81	stage iii	2965	1
TCGA-E2-A15F	High Risk	64	stage i	658	0
TCGA-E2-A10F	High Risk	47	stage ii	878	0

TCGA-E9-A226	High Risk	45	stage iii	1048	1
TCGA-GM-A3NY	High Risk	72	stage ii	1162	0
TCGA-A2-A0YI	High Risk	62	stage i	1505	0
TCGA-D8-A73X	High Risk	53	stage ii	767	0
TCGA-A2-A25A	High Risk	44	stage ii	3276	0
TCGA-AO-A0JE	High Risk	53	stage iii	2335	0
TCGA-BH-A0H7	High Risk	65	stage iii	702	0
TCGA-A8-A06P	High Risk	63	stage iii	396	0
TCGA-A7-A0DA	High Risk	62	stage ii	1085	0
TCGA-E2-A15K	High Risk	58	stage ii	275	0
TCGA-E9-A1RE	High Risk	74	stage iii	1419	0
TCGA-A7-A3RF	High Risk	79	stage ii	408	0
TCGA-D8-A1X9	High Risk	66	stage ii	727	0
TCGA-S3-AA17	High Risk	64	stage ii	424	0
TCGA-C8-A26V	High Risk	47	stage iii	616	0
TCGA-A7-A0DB	High Risk	56	stage ii	1007	0
TCGA-AO-A0JL	High Risk	59	stage iii	1683	0
TCGA-D8-A1XO	High Risk	56	stage ii	1682	0
TCGA-BH-A0HO	High Risk	48	stage ii	76	0
TCGA-E9-A247	High Risk	59	stage i	1186	0
TCGA-A8-A07F	High Risk	65	stage ii	577	0
TCGA-D8-A27I	High Risk	58	stage iii	439	0
TCGA-B6-A0IE	High Risk	38	stage iii	1993	1
TCGA-BH-A0RX	High Risk	59	stage ii	170	0
TCGA-A2-A0D4	High Risk	37	stage ii	767	0
TCGA-OL-A66J	High Risk	80	stage i	1996	0
TCGA-AQ-A04H	High Risk	61	stage iii	754	0
TCGA-E9-A1NA	High Risk	58	stage ii	1112	0
TCGA-GM-A2DF	High Risk	53	stage ii	2155	0

TCGA-GI-A2C9	High Risk	58	stage ii	3342	0
TCGA-BH-A0BG	High Risk	73	stage i	1871	0
TCGA-BH-A0DG	High Risk	30	stage ii	2041	0
TCGA-BH-A0DI	High Risk	63	stage ii	912	0
TCGA-A7-A0CJ	High Risk	57	stage ii	931	0
TCGA-LL-A5YP	High Risk	49	stage ii	450	0
TCGA-AO-A126	High Risk	39	stage ii	3307	0
TCGA-A7-A5ZX	High Risk	48	stage iii	336	0
TCGA-LL-A9Q3	High Risk	69	stage iii	532	0
TCGA-B6-A1KC	High Risk	67	stage ii	1326	0
TCGA-E2-A14V	High Risk	53	stage ii	1042	0
TCGA-A7-A0DB	High Risk	56	stage ii	1007	0
TCGA-BH-A0B1	High Risk	66	stage ii	1148	0
TCGA-AC-A3W5	High Risk	65	stage ii	504	0
TCGA-AR-A24H	High Risk	65	stage ii	4894	0
TCGA-E2-A14O	High Risk	76	stage iii	1359	0
TCGA-A8-A075	High Risk	42	stage ii	518	0
TCGA-OL-A5RW	High Risk	40	stage ii	1106	0
TCGA-B6-A1KF	High Risk	68	stage ii	3088	0
TCGA-PE-A5DD	High Risk	64	stage ii	1953	0
TCGA-A2-A0YH	High Risk	53	stage iii	659	0
TCGA-E2-A1IL	High Risk	78	stage ii	118	0
TCGA-E2-A1LL	High Risk	73	stage iii	1309	0
TCGA-AR-A2LQ	High Risk	59	stage ii	1233	0
TCGA-OL-A66L	High Risk	71	stage i	1301	0
TCGA-A8-A08F	High Risk	59	stage iii	1004	0
TCGA-A8-A07G	High Risk	65	stage ii	577	0
TCGA-AO-A12D	High Risk	43	stage ii	2515	0
TCGA-BH-A0HF	High Risk	77	stage i	727	0

TCGA-AO-A03L	High Risk	34	stage iii	2442	0
TCGA-A8-A097	High Risk	65	stage ii	365	0
TCGA-BH-A0B8	High Risk	64	stage i	1569	0
TCGA-AO-A0J6	High Risk	61	stage ii	1140	0
TCGA-B6-A0IG	High Risk	50	stage ii	4456	1
TCGA-BH-A8FZ	High Risk	58	stage i	574	0
TCGA-BH-A0BC	High Risk	60	stage iii	974	0
TCGA-D8-A1Y0	High Risk	65	stage iii	472	0
TCGA-AO-A128	High Risk	61	stage ii	3248	0
TCGA-AR-A1AU	High Risk	39	stage iii	2868	0
TCGA-BH-A42V	High Risk	41	stage i	635	0
TCGA-B6-A0I9	High Risk	62	stage iv	362	1
TCGA-EW-A1PH	High Risk	52	stage ii	607	0
TCGA-EW-A1OY	High Risk	63	stage ii	908	0
TCGA-EW-A1P6	High Risk	64	stage ii	562	0
TCGA-AC-A7VB	High Risk	51	stage ii	250	0
TCGA-LL-A441	High Risk	62	stage i	996	0
TCGA-A7-A0DC	High Risk	63	stage i	906	0
TCGA-A2-A0SY	High Risk	62	stage iii	1347	0
TCGA-E2-A1LB	High Risk	41	stage ii	2306	0
TCGA-D8-A147	High Risk	45	stage i	584	0
TCGA-EW-A3E8	High Risk	60	stage ii	1035	0
TCGA-LQ-A4E4	High Risk	73	stage iii	849	0
TCGA-A8-A08G	High Risk	41	stage ii	607	0
TCGA-AO-A03P	High Risk	54	stage ii	2911	1
TCGA-E2-A1IE	High Risk	61	stage ii	2362	0
TCGA-EW-A2FS	High Risk	41	stage ii	1604	0
TCGA-B6-A0WY	High Risk	40	stage iii	3461	1
TCGA-A2-A04T	High Risk	62	stage ii	2246	0

TCGA-A2-A04Y	High Risk	53	stage ii	1099	0
TCGA-AO-A12C	High Risk	42	stage ii	2372	0
TCGA-AR-A1AO	High Risk	47	stage ii	2618	0
TCGA-BH-A0AU	High Risk	45	stage ii	1914	0
TCGA-OL-A5RV	High Risk	43	stage iii	1062	0
TCGA-BH-A0DQ	High Risk	42	stage ii	98	0
TCGA-E2-A573	High Risk	48	stage i	1062	0
TCGA-AR-A1AJ	High Risk	83	stage i	2383	0
TCGA-HN-A2OB	High Risk	45	stage ii	1900	1
TCGA-GM-A2DB	High Risk	62	stage ii	2406	0
TCGA-E2-A15K	High Risk	58	stage ii	275	0
TCGA-E9-A295	High Risk	71	stage ii	375	0
TCGA-AR-A1AX	High Risk	64	stage i	2629	0
TCGA-AR-A1AS	High Risk	54	stage ii	1150	0
TCGA-AO-A0J3	High Risk	67	stage ii	651	0
TCGA-AR-A24L	High Risk	26	stage ii	2866	1
TCGA-A2-A4RY	High Risk	46	stage iii	648	0
TCGA-AR-A1AL	High Risk	60	stage iii	2971	0
TCGA-AQ-A7U7	High Risk	55	stage iii	584	1
TCGA-E2-A15E	High Risk	40	stage ii	630	0
TCGA-A8-A0A6	High Risk	64	stage iii	640	0
TCGA-AR-A5QN	High Risk	68	stage iii	1013	0
TCGA-A2-A3KC	High Risk	55	stage ii	1102	0
TCGA-A8-A09E	High Risk	73	stage iii	1492	0
TCGA-XX-A899	High Risk	46	stage iii	467	0
TCGA-D8-A1XD	High Risk	36	stage iii	522	0
TCGA-E2-A14X	High Risk	55	stage iii	972	0
TCGA-LL-A5YO	High Risk	50	stage i	440	0
TCGA-A8-A07O	High Risk	51	stage ii	304	0

TCGA-AC-A5XU	High Risk	74	stage ii	455	0
TCGA-A2-A4S1	High Risk	66	stage ii	820	0
TCGA-AR-A0TT	High Risk	53	stage iii	3316	0
TCGA-B6-A1KN	High Risk	57	stage iii	4233	0
TCGA-E2-A15S	High Risk	34	stage ii	428	0
TCGA-E2-A574	High Risk	44	stage ii	1179	0
TCGA-A2-A0YJ	High Risk	39	stage iii	566	0
TCGA-AR-A1AV	High Risk	68	stage ii	1864	0
TCGA-AC-A3YI	High Risk	74	stage iii	707	0
TCGA-A2-A0SV	High Risk	63	stage iv	825	1
TCGA-A7-A0CG	High Risk	78	stage ii	1043	0
TCGA-LL-A5YL	High Risk	64	stage ii	519	0
TCGA-BH-A0HP	High Risk	65	stage iii	414	0
TCGA-AR-A24Q	High Risk	49	stage ii	3172	0
TCGA-D8-A27W	High Risk	55	stage iii	373	0
TCGA-A7-A6VX	High Risk	68	stage ii	317	0
TCGA-E9-A22E	High Risk	56	stage iii	1269	0
TCGA-E2-A14P	High Risk	79	stage iii	1246	0
TCGA-OL-A66H	High Risk	74	stage i	812	0
TCGA-E2-A1LI	High Risk	57	stage ii	3121	0
TCGA-A7-A4SC	High Risk	62	stage ii	446	0
TCGA-E9-A1RG	High Risk	62	stage iii	647	0
TCGA-E2-A14Z	High Risk	64	stage i	563	1
TCGA-A7-A426	High Risk	50	stage iii	364	0
TCGA-EW-A3U0	High Risk	61	stage iii	532	0
TCGA-BH-A1EV	High Risk	45	stage iii	365	1
TCGA-E2-A10B	High Risk	67	stage ii	1141	0
TCGA-A2-A1G1	High Risk	85	stage ii	584	0
TCGA-AC-A3OD	High Risk	68	stage ii	451	0

TCGA-D8-A1JM	High Risk	59	stage ii	590	0
TCGA-AR-A2LK	High Risk	62	stage iii	1649	1
TCGA-LL-A5YN	High Risk	46	stage ii	447	0
TCGA-BH-A18H	High Risk	63	stage i	652	0
TCGA-AC-A6IX	High Risk	49	stage iii	373	0
TCGA-A8-A08S	High Risk	71	stage ii	1004	0
TCGA-BH-A0B9	High Risk	44	stage i	1572	0
TCGA-A2-A1FW	High Risk	62	stage iii	528	0
TCGA-AO-A1KS	High Risk	69	stage ii	350	0
TCGA-BH-A0DX	High Risk	62	stage i	2156	0
TCGA-B6-A3ZX	High Risk	50	stage iv	1152	1
TCGA-E9-A2JT	High Risk	63	stage ii	288	0
TCGA-A2-A3XV	High Risk	46	stage ii	996	0
TCGA-AR-A1AT	High Risk	62	stage ii	1272	1
TCGA-GM-A2D9	High Risk	69	stage i	1812	1
TCGA-BH-A18F	High Risk	50	stage ii	1001	0
TCGA-BH-A0HU	High Risk	52	stage i	392	0
TCGA-AC-A3TM	High Risk	50	stage iii	762	0
TCGA-A8-A09B	High Risk	58	stage iii	365	0
TCGA-E2-A154	High Risk	68	stage i	591	0
TCGA-BH-A0BP	High Risk	76	stage i	2296	1
TCGA-A7-A26I	High Risk	65	stage ii	661	0
TCGA-A8-A08X	High Risk	43	stage iii	1308	0
TCGA-E9-A2JS	High Risk	72	stage ii	904	1
TCGA-A8-A084	High Risk	81	stage ii	458	0
TCGA-XX-A89A	High Risk	68	stage ii	488	0
TCGA-BH-A0GY	High Risk	67	stage ii	923	0
TCGA-BH-A0BW	High Risk	71	stage i	2371	0
TCGA-AR-A0TX	High Risk	64	stage ii	1972	0

TCGA-BH-A0BD	High Risk	47	stage ii	554	0
TCGA-E2-A1BD	High Risk	53	stage ii	1133	0
TCGA-AR-A5QP	High Risk	54	stage ii	1185	0
TCGA-A2-A3XY	High Risk	49	stage ii	1093	1
TCGA-A2-A0SW	High Risk	82	stage iv	1365	1
TCGA-E2-A15A	High Risk	45	stage iii	710	0
TCGA-A2-A0T0	High Risk	59	stage ii	533	0
TCGA-E9-A1RA	High Risk	48	stage i	1369	0
TCGA-E2-A15A	High Risk	45	stage iii	710	0
TCGA-A7-A26E	High Risk	71	stage iii	954	0
TCGA-A8-A09D	High Risk	47	stage ii	1522	0
TCGA-A7-A26E	High Risk	71	stage iii	954	0
TCGA-A2-A25F	High Risk	66	stage ii	322	0
TCGA-E2-A3DX	High Risk	43	stage iii	1325	0
TCGA-D8-A1Y3	High Risk	61	stage iii	430	0
TCGA-A7-A13D	High Risk	46	stage ii	965	0
TCGA-A2-A0EN	High Risk	70	stage ii	4088	0
TCGA-3C-AALI	High Risk	50	stage ii	4005	0
TCGA-OL-A5S0	High Risk	66	stage ii	620	0
TCGA-E2-A10C	High Risk	54	stage ii	1220	0
TCGA-BH-A0H9	High Risk	69	stage ii	1247	0
TCGA-A2-A0CL	High Risk	37	stage iii	3015	0
TCGA-BH-A0BR	High Risk	59	stage i	2330	0
TCGA-B6-A0WW	High Risk	58	stage iv	558	1
TCGA-AC-A23E	High Risk	50	stage ii	698	0
TCGA-AR-A252	High Risk	50	stage i	2838	0
TCGA-D8-A3Z6	High Risk	56	stage iii	563	0
TCGA-E9-A1RH	High Risk	63	stage ii	1417	0
TCGA-AO-A0J5	High Risk	48	stage iv	792	1

TCGA-A7-A4SF	High Risk	54	stage ii	545	0
TCGA-E2-A15R	High Risk	64	stage ii	1732	0
TCGA-A7-A26F	High Risk	55	stage i	738	0
TCGA-AC-A2QI	High Risk	76	stage iii	588	0
TCGA-A8-A06X	High Risk	77	stage ii	943	1
TCGA-E9-A1R7	High Risk	64	stage ii	1467	0
TCGA-D8-A13Z	High Risk	51	stage i	635	0
TCGA-AR-A0TS	High Risk	46	stage ii	2558	0
TCGA-A8-A06R	High Risk	69	stage ii	547	0
TCGA-E2-A1IN	High Risk	60	stage i	675	0
TCGA-BH-A0DL	High Risk	64	stage ii	2381	0
TCGA-A2-A0EQ	High Risk	64	stage ii	2426	0
TCGA-BH-A0C1	High Risk	61	stage iii	1411	1
TCGA-AC-A6IX	High Risk	49	stage iii	373	0
TCGA-D8-A27R	High Risk	41	stage iii	307	0
TCGA-OL-A5D8	High Risk	40	stage ii	973	0
TCGA-AR-A5QM	High Risk	62	stage ii	2231	0
TCGA-BH-A0BZ	High Risk	59	stage iii	2255	0
TCGA-A2-A0D1	High Risk	76	stage ii	1051	0
TCGA-AR-A24T	High Risk	46	stage iii	3202	0
TCGA-A7-A0DC	High Risk	63	stage i	906	0
TCGA-D8-A1JG	High Risk	62	stage ii	1612	0
TCGA-AC-A2B8	High Risk	84	stage ii	677	0
TCGA-LL-A6FR	High Risk	50	stage ii	489	0
TCGA-A2-A0YT	High Risk	56	stage iii	723	1
TCGA-B6-A0RT	High Risk	39	stage iii	2721	0
TCGA-A7-A2KD	High Risk	53	stage iii	679	0
TCGA-E2-A10E	High Risk	64	stage ii	865	0
TCGA-BH-A0B6	High Risk	47	stage i	2483	0

TCGA-A8-A092	High Risk	48	stage iii	942	0
TCGA-AR-A24S	High Risk	61	stage i	2976	0
TCGA-AR-A255	High Risk	62	stage i	2161	0
TCGA-A2-A0YG	High Risk	63	stage iii	666	0
TCGA-GM-A2DA	High Risk	46	stage ii	6593	1
TCGA-A2-A0EY	High Risk	62	stage ii	1925	0
TCGA-E2-A1LE	High Risk	71	stage iii	879	1
TCGA-AR-A5QQ	High Risk	68	stage iii	322	1
TCGA-E2-A105	High Risk	79	stage ii	1308	0
TCGA-A2-A1FV	High Risk	74	stage ii	714	0
TCGA-BH-A202	High Risk	60	stage ii	795	0
TCGA-AR-A24U	High Risk	47	stage ii	3128	0
TCGA-EW-A423	High Risk	75	stage ii	533	0
TCGA-E2-A1B0	High Risk	50	stage iii	1631	0
TCGA-C8-A3M7	High Risk	60	stage iii	1034	1
TCGA-D8-A1X5	High Risk	81	stage iii	565	0
TCGA-A8-A06Y	High Risk	66	stage ii	791	0
TCGA-D8-A1JJ	High Risk	54	stage ii	611	0
TCGA-A8-A08P	High Risk	70	stage iii	943	0
TCGA-E2-A159	High Risk	50	stage ii	762	0
TCGA-E9-A1N6	High Risk	52	stage ii	678	1
TCGA-AC-A5EH	High Risk	76	stage ii	511	0
TCGA-BH-A0E6	High Risk	69	stage i	293	0
TCGA-BH-A0B7	High Risk	42	stage ii	2559	0
TCGA-D8-A1XK	High Risk	55	stage ii	441	0
TCGA-A8-A08B	High Risk	52	stage ii	1156	0
TCGA-D8-A1JA	High Risk	60	stage ii	502	0
TCGA-A2-A0CX	High Risk	52	stage ii	1728	0
TCGA-D8-A1JC	High Risk	59	stage iii	480	0

TCGA-E2-A2P5	High Risk	78	stage iii	821	1
TCGA-AO-A0JD	High Risk	59	stage iii	2190	0
TCGA-A8-A08L	High Risk	89	stage iii	30	1
TCGA-OL-A66I	High Risk	36	stage ii	714	0
TCGA-AR-A251	High Risk	51	stage iii	3030	0
TCGA-AC-A2BK	High Risk	78	stage iii	2222	0
TCGA-AR-A0U2	High Risk	47	stage iii	2551	1
TCGA-BH-A1EV	High Risk	45	stage iii	365	1
TCGA-EW-A2FR	High Risk	59	stage iii	1673	0
TCGA-A7-A13D	High Risk	46	stage ii	965	0
TCGA-A2-A0YM	High Risk	67	stage ii	965	0
TCGA-D8-A1XZ	High Risk	81	stage iii	466	0
TCGA-E2-A15M	High Risk	66	stage ii	336	1
TCGA-AC-A2QH	High Risk	58	stage ii	1005	0

Table S2 Spearman correlations between the mRNA level of five key immune checkpoints, and between risk score and the mRNA level.

	Riskscore	LAG3	TIGIT	CTLA4	PD_L1	TIM3
Riskscore	1	0.198586	0.1454817	0.1697502	0.0641392	0.0942833
LAG3	0.198586	1	0.7569961	0.7912102	0.6351957	0.5262816
TIGIT	0.1454817	0.7569961	1	0.9107397	0.7154985	0.6274906
CTLA4	0.1697502	0.7912102	0.9107397	1	0.7030872	0.6077382
PD_L1	0.0641392	0.6351957	0.7154985	0.7030872	1	0.6801158
TIM3	0.0942833	0.5262816	0.6274906	0.6077382	0.6801158	1
